# Supplementary material for: Pro-inflammation and clinical correlates of unsuppressed HIV-viral load in children living with perinatally-acquired HIV 1 in Zambia
Source: PLoS One. 2025 Oct 9;20(10):e0334109. doi: 10.1371/journal.pone.0334109 (PMC12510508; doi:10.1371/journal.pone.0334109)
Supplement: Attachment — Submitted filename: PONE-D-24-50031R1 Re-review R1 - 19th Sept 2025.docx [file pone.0334109.s004.docx]

**PONE-D-24-50031R1** **Re-review R1: 19^th^ September 2025**

The authors have addressed the comments and suggestions raised in the initial manuscript.

However, I have the following minor edits;

Introduction

1. Line 63: …..HIV clinic at Arthur DAVISON Children’s Hospital (ADH). – Davison is omitted.

Methods

1. Line 67: use abbreviation – ADH as above.

Results

2. Line 144: Arthur Davison Children’s Hospital is already abbreviated as ADH.

Discussion

1. Line 235: Delete “one” full stop.
2. Line 251: ----viral load, abbreviate as VL
